# Cost of illness in inclusion body myositis: results from a cross-sectional study in Germany

**DOI:** 10.1186/s13023-023-02902-3

**Published:** 2023-10-25

**Authors:** Katja C. Senn, Simone Thiele, Karsten Kummer, Maggie C. Walter, Klaus H. Nagels

**Affiliations:** 1 Chair of Healthcare Management and Health Services Research, University of Bayreuth, Parsifalstrasse 25, 95445 Bayreuth, Germany; 2 Department of Neurology, Friedrich Baur Institute, LMU University Hospital, LMU Munich, Ziemssenstrasse 1, 80336 Munich, Germany; 3 Department of Neurology, University Medical Center Goettingen, 37075 Göttingen, Germany

**Keywords:** Inclusion body myositis, Cost of illness, Direct costs, Indirect costs, Informal care costs, Neuromuscular disease, Health services research

## Abstract

**Background:**

Inclusion body myositis (IBM) is the most frequent type of myositis in elder patients with a slow chronic progression and refractory to treatment. Previous cost of illness (COI) studies in IBM used claims data to estimate direct costs in the US. No evidence exists globally on both direct and indirect costs in IBM from a societal perspective. We conducted a survey in patients registered in the German IBM patient registry. Self-developed items were used to assess the utilized healthcare resources and estimate the cost. The German Self-Administered Comorbidity Questionnaire (SCQ-D), the sIBM Physical Functioning Assessment (sIFA) and patient-reported measures for satisfaction and improvements in healthcare were applied for an explorative analysis.

**Results:**

In total, 82 patients completed the survey. We estimated the mean total annual per capita COI of US$102,682 (95% CI US$82,763–US$123,090) in 2021. 92.7% of the total COI were direct costs. Medical costs were similar to nonmedical costs, with substantial costs for pharmacotherapy and informal care. Depending on the prevalence estimate, the total national COI per year were US$42.7 million–US$213.7 million. Significant differences in total COI were identified for the degree of disability, marital and employment status (*p* < 0.05).

**Conclusions:**

We identified remarkable and heterogenous cost in IBM. As informal care costs represented the most relevant cost driver, caregiver burden is a major factor in the patient journey. For the first time, comprehensive economic potentials were identified as a basis to improve the actual care situations and prioritizing future activities for research, pharmaceutical and digital product development as well as health politics.

**Supplementary Information:**

The online version contains supplementary material available at 10.1186/s13023-023-02902-3.

## Background

Inclusion body myositis (IBM) is an idiopathic inflammatory myopathy (IIM) with a chronic and slowly progressive course. A high variability in prevalence rates has been reported of 1–71 per million (e.g., 1.06 per million in Turkey [1] or 70.6 per million in the United States (US) [2]), up to 139 per million [3] in elder patients aged 50 and older in South Australia [4–6]. The main clinical patterns of IBM are a late-onset asymmetric muscle weakness, mostly affecting the quadriceps femoris, wrist and finger flexors, tibialis anterior and hip flexors [7–9]. Weakness in pharyngeal muscles leads to dysphagia in up to 80% of patients during progression; aspiration pneumonia, dehydration or malnutrition can cause increased morbidity and mortality [10, 11]. Thus far, causative treatments, which are directed against the cause of IBM, are not available [12, 13]. Physiotherapy, symptomatic treatment of dysphagia and therapeutic attempts with intravenous immunoglobulins (IVIg) are recommended for clinical practice [12, 14]. Impaired health-related quality of life (HRQoL), caregiver burden as well as the need for expensive assistive devices or housing modifications suggest a high risk for overall disease burden in IBM patients [15–17]. Recent studies examined the cost of illness (COI) and healthcare utilization in IBM either only for the US population or explicitly excluded IBM patients in COI studies and focused on IIM patients [18–21]. In addition to the reported substantial disease burden for the US, financial distress has also been suggested in a Japanese IBM population [16]. This underpins the relevance and need of COI transparency along IBM patient care trajectories. Correspondingly, this enables informed resource allocation decisions for finite healthcare resources and a more equitable adjustment of co-payments or disease related private spendings [16, 18, 20, 22]. Comprehensive real-world data regarding COI, patient-reported outcomes (PRO) and experiences (PRE) are sparse in rare and neuromuscular diseases (NMD), but are already an integral part of regulatory frameworks for benefit assessments of future therapies or treatment approaches to demonstrate the effectiveness and actual patient-centred perceptions [23–28]. To the best of our knowledge and according to a scoping review of García‑Pérez et al. from the year 2021 [25], no COI study has focused on both direct as well as indirect costs in IBM integrating PRO and PRE. García‑Pérez et al. analysed thereby 63 COI studies on 42 different rare diseases in 25 countries [25].

Accordingly, the aim of this study is twofold: first, it is to assess the direct and indirect costs per year in IBM in Germany for the reference year 2021 from a societal perspective. Second, it is to identify and understand the impact of potential cost driving factors.

## Methods

### Study population and study design

Over 14 weeks between June and October 2021 we conducted a quantitative cross-sectional study as second part of a mixed-methods design to explore the complex patient-reported care situation of IBM in the German healthcare setting. Patients from the German IBM patient registry (www.IBM-registry.org) were eligible, if they were diagnosed with probable or certain IBM [29, 30] and were German-speaking. Patients within the patient registry are diagnosed according to recent European neuromuscular centre (ENMC) criteria [29], the register has been available for registration since 2016. Patients living in other countries than Germany and not utilizing resources from the German health system or those not German-speaking were ineligible. A questionnaire from our previous COI studies in other NMD was adapted to disease specific characteristics of IBM according to the results of our systematic review about HRQoL in IBM [15], results of our exploratory interviews (*n* = 8) about HRQoL and the care situation in IBM in the German healthcare system (first part of the mixed-methods design; article under review) as well as with expert guidance from neurologists, physiotherapists, health economists and health services researchers [31–33]. The involved clinicians and therapists provided expert knowledge about the actual care situations of IBM patients in the German healthcare setting. The health economists and health services researchers searched for reported resource utilization in IBM in the literature. We adapted the questionnaire mainly regarding disease specific therapies (e.g., adding IVIg treatment), aids (e.g., adding care aids for hand motor function) and patient characteristics (e.g., excluding previous survey parts for parents, as IBM is not a disease of the childhood). The survey included a set of questions on sociodemographic variables, PRO and PRE measures (PROMs, PREMs) as well as the utilization of healthcare resources and other disease-specific private payments. To capture all IBM disease specific resources, a pre-test with patients and exploratory interviews on the care situation and HRQoL was conducted. The ENMC diagnostic criteria were gathered through the registry items [29, 30]. Patients were given the option to complete an electronic version of the survey via Qualtrics (www.qualtrics.com) or on paper. All patients gave their written consent with the option of withdrawal to participate. Ethical approval was obtained from the ethics board of the Ludwig-Maximilians-University of Munich.

### Cost assessment

A micro-costing (bottom-up) approach was applied. We retrospectively assessed the mean direct and indirect costs in IBM from a societal perspective for 1 year, extrapolating the costs in the recall periods of 3, 6, 12 or 24 months. A constant resource utilization was assumed for total COI in this prevalence approach [34].

Direct costs comprise the costs for resource utilization in the health care sector during health care provision (medical costs) and the costs for resource utilization to support the production of medical services in the health sector (nonmedical costs). To calculate the direct medical COI, the resources (e.g., medication, inpatient and outpatient visits, psychological support) were valued with the latest price lists of 2021 for Germany (reference date: 05.05.2022) or with inflated prices to the year 2021 using the harmonized index of consumer prices [35, 36]. The nonmedical direct COI were estimated through patient-reported costs (e.g., modifications at home or at work), whereby informal care and travel costs were assessed with recommended unit prices [35, 37–40]. To respect the national reimbursement situation of the utilized resources, we used the latest health economic recommendations for cost assessment in the German healthcare setting and definitions of costs by the Institute for Quality and Efficiency in Health Care (IQWiG) [31], Krauth 2010 [35] and Bock et al. 2015 [33], respectively. Additional file 1 shows a detailed overview of the applied data sources and unit prices and Table 3 summarizes the reported included costs below.

We used the human capital approach to calculate the indirect COI, defined as productivity losses due to IBM (sick leaves, part-time employment, unemployment and early retirement). Self-reported wages in the year 2021 served as a basis for this estimation.

To determine the economic burden of IBM in Germany, we used two different prevalence estimates according to Rath and Yamazaki [41] and Callan et al. [6] (Table 5). We assumed as a minimum 416 up to 2,081 affected people with IBM in Germany in the year 2021. To our knowledge, specified prevalence data of IBM have not been published for Germany.

### Patient-reported outcome and experience measures

To contrast the COI with patient-relevant variables, we explored the role of physical functioning in IBM and self-reported comorbidities, applying the German version of sIBM Physical Functioning Assessment (sIFA) [42] and the German Self-Administered Comorbidity Questionnaire (SCQ-D) [43, 44], respectively.

The sIFA is a 0–10 numerical rating scale instrument (0 = no difficulty, 10 = unable to do) with 11 items. Patients rate their difficulties in swallowing, lower and upper body functioning as well as general functioning over the last 7 days [45]. To date, the sIFA is the only PROM for IBM in accordance with the US Food and Drug Administration’s (FDA) PRO guidelines [46].

The SCQ-D evaluates the extent and type of comorbidities. Patients specify for 13 predefined health problems in a binary form, if they have the problem, if they get treatment and if the health problem causes an impairment in their activities of daily living. The maximum score is 39 points (0–3 for each health problem) [44].

Furthermore, participants rated their satisfaction with healthcare and health insurance on a 5-point scale (worst to best satisfaction: 1–5) [47]. A free text box was used to explicate qualitative suggestions for improvements in IBM healthcare.

## Statistical analysis

All data were analysed with the IBM^©^ SPSS^©^ Statistics version 28 (IBM, Armonk, New York, US). The significance level was set to 5%. The mean COI were calculated in euros and converted into US dollars (1US$ = 0.74€; reference year 2021) [48]. Missing data on PROMs and PREMs were excluded (one case with the sIFA). The use of single imputation was planned for other variables than PROMs and PREMs. Ultimately, single imputation did not have to be applied due to the completeness of the data. As the Shapiro–Wilk test indicated no normally distributed data, the 95% confidence intervals (CIs) were calculated with bias corrected and accelerated bootstrapping method (1000 parameter estimates). As our analysis was not hypothesis-driven and showed large standard deviations, we used the Bonferroni correction to prevent Type I error inflation [49]. For the descriptive and econometric analysis, we applied the Friedman test, Mann–Whitney U test, Kruskal–Wallis test and Spearman’s correlation coefficient.

The Checklist for the Development and Assessment of Cost of Illness Studies [50] guided this research report.

## Results

### Patient characteristics

From 111 invited patients, a total of 82 patients completed the survey (response rate 74%). Table 1 presents the sociodemographic and health-related characteristics of this sample. Most patients were male (78%); the median age was 71 years (range 53–84). The median age at the time of symptom onset was 58 years, and 63.5 years at the diagnosis, respectively. 35 patients were diagnosed firstly with other diseases than IBM, mainly with polymyositis or amyotrophic lateral sclerosis (each *n* = 7); the median duration from getting a misdiagnosis to a correct IBM diagnosis was 24 months. The majority in this sample (43.9%) had a clinico-pathologically defined IBM diagnosis according to ENMC criteria [29] (34.1% clinically; 22% probable, respectively). In comparison to the typical distributions in the German population, our sample showed a lower percentage of statutory health insurance (67.5% in our sample vs. 73.4% of the general population [51]) and a higher educational level as well as a comparable distribution of the geographic residence in East and West Germany [51, 52]. Most of the patients were retired (80.2%) and lived together with their spouse (81.7%). Four patients (4.9%) lived in a nursing home, whereas 63.4% of all participants had a care level of 2–5 (general range of German care levels: 1–5) and nearly half the patients (49.4%) were classified into a degree of disability of 80–100 (general range of German degree of disability: 20–100). One fifth of the patients had moved at least once due to inaccessible flats or houses. The majority experienced at least some (30.4%), quite a bit (17.7%) or very high financial difficulties (11.4%) due to IBM.

**Table 1:** Patient sociodemographic and health-related characteristics (n = 82)

Characteristics	n (%) or median (IQR)
Male	64 (78.0)
Age	71 (65–78)
Age groups	
< 65	19 (23.2)
65–69	13 (15.9)
70–74	20 (24.4)
75–79	15 (18.3)
> 80	15 (18.3)
Age at symptom onset (n = 81)	58 (52–64)
Age at diagnosis	63.5 (58–69)
Duration from other diagnoses until IBM diagnosis in months (n = 35)	24 (6–48)
ENMC criteria^a^	
Clinico-pathologically defined	36 (43.9)
Clinically defined	28 (34.1)
Probable	18 (22.0)
BMI (n = 81)^b^	
Underweight	0 (0)
Normal weight	37 (45.7)
Overweight	36 (44.4)
Obese	8 (9.9)
Marital status	
Single	1 (1.2)
Widowed	6 (7.3)
Divorced	6 (7.3)
Married, living apart	2 (2.4)
Married, living together	67 (81.7)
German geographic location^c^	
West	73 (89.0)
East	9 (11.0)
Employment status (n = 81)	
Retired	65 (80.2)
Non-working due to IBM	2 (2.5)
Employed	9 (11.1)
Self-employed	5 (6.2)
Educational level (n = 80)^d^	
Low	1 (1.3)
Medium	46 (57.5)
High	33 (41.3)
Housing conditions	
Flat	28 (34.1)
House	47 (57.3)
Home for the elderly	1 (1.2)
Assisted living	2 (2.4)
Nursing home	4 (4.9)
Removals due to IBM	16 (19.5)
Reasons for removals due to IBM (n = 16)^e^	
No accessibility	15 (93.8)
Subsistence not possible	10 (62.5)
Removal recommended by others	8 (50.0)
Independent housework not possible	7 (43.8)
High caregiver burden	3 (18.8)
Loneliness	2 (12.5)
Financial difficulties due to IBM (n = 79)	
Not	32 (40.5)
A little	24 (30.4)
Quite a bit	14 (17.7)
Very	9 (11.4)
Statutory health insurance (n = 80)	54 (67.5)
Care level^f^	
No care level	30 (36.6)
Care level 1	0
Care level 2	18 (22.0)
Care level 3	20 (24.4)
Care level 4	9 (11)
Care level 5	5 (6,1)
Degree of disability^g^ (n = 77)	
No degree of disability	12 (15.6)
20–40	6 (7.8)
50–70	21 (27.3)
80–100	38 (49.4)

*BMI* body mass index, *IQR* interquartile range. Percentages may not total 100 due to rounding

^a^Data gathered from IBM patient registry

^b^BMI categories refer to WHO definition [74]

^c^East Germany includes Berlin and the five re-established states of the former German Democratic Republic. In the case of missing values, the reference value for the population is given (n =)

^d^Educational level is reported according to the International Standard Classification of Education (ISCED) 2011 [75]

^e^Multiple response item, cumulative value

^f^In 2017, the definition of the need for care was revised in Germany. The extent of benefits from the German statutory care insurance are based on an individual score within six life domains of a person. A higher care level indicates a worse state of independence and capabilities [76]

^g^The degree of disability (20–100) is graduated in steps of 10. A higher degree indicates a higher level of physical, psychological or social disability [77]

### Patient-reported outcome and experience measures

Table 2 illustrates the data on patient-reported outcome- and experience measures. The self-reported physical function using the sIFA showed in total a median of 74 (42–88 IQR). The item with the highest median was difficulty ‘get up from the floor’ (10) and the lowest median was reported for the item difficulty ‘swallow liquids’ (1). Within the ENMC categories, the highest median of the sIFA of 76 was identified in patients with a clinico-pathologically defined IBM, followed by a median of 73 in clinically defined IBM and lastly a median of 43 in probable defined IBM. The differences of physical function using the sIFA within the ENMC criteria were not significant (*p* = 0.240). These differences might not be explained through age, as the median age only differed slightly and not significantly within the ENMC categories (data not shown; clinico-pathological: 71; clinically: 74; probable: 70).

**Table 2:** PROMs and PREMs: physical functioning, comorbidities and satisfaction with healthcare

	Median (IQR) or n (%)	Possible range
sIFA total (n = 81)	74 (42–88)	0–110
Stand from ordinary chair	8 (4–10)	0–10
Get up from the floor	10 (8–10)	0–10
Get on and off toilet	8 (5–10)	0–10
Walk on a flat, firm surface	5 (3–8)	0–10
Walk outdoors	8 (5–10)	0–10
Go up or down 5 steps	9 (4–10)	0–10
Step up and down curbs	8 (4–10)	0–10
Swallow liquids	1 (0–3)	0–10
Swallow solids	2 (0–5)	0–10
Carry a 5-pound object	6 (3–10)	0–10
Grip and use small objects	6 (3–9)	0–10
SCQ-D (n = 82)	5 (2–8)	0–39
Problem	2.5 (1–4)	0–13
Treatment	1.5 (1–3)	0–13
Limited activities	0 (0–1)	0–13
Satisfaction with healthcare (n = 81)		
Very	22 (27.2)	
Quite a bit	34 (42.0)	
Moderately	18 (22.2)	
A little	5 (6.2)	
Not	2 (2.5)	
Satisfaction with health insurance (n = 82)		
Very	23 (28.0)	
Quite a bit	37 (45.1)	
Moderately	10 (12.2)	
A little	10 (12.2)	
Not	2 (2.49)	

*IQR* interquartile range, *sIFA* sIBM physical functioning assessment, *SCQ-D* German self-administered comorbidity questionnaire. Percentages may not total 100 due to rounding

Patients reported a median of 2.5 comorbidities in the SCQ-D, mainly high blood pressure (57.3%), back pain (42.7%), arthritis (30.5%) and heart disease (28%; data not shown). The median for received corresponding treatments was lower (1.5). The greatest differences between reporting a comorbidity as a problem, but not receiving treatment, were observed for back pain (19.5%), arthritis (19.5%), ulcer or stomach disease (12.2%) and depression (11%). Although patients reported in total a median of 0 for the limitation of activities due to their comorbidities. Limited activities were mainly experienced due to back pain (26.8%), arthritis (17.1%), ulcer or stomach disease (12.2%) and depression (8.5%).

We identified no significant (*p* = 0.088) intra-individual differences regarding the satisfaction with healthcare providers and healthcare insurance. Overall, most of the patients were quite a bit (42%; 45.1%) or very satisfied (27.2%; 28%) with their healthcare providers and insurance, respectively. Moreover, suggestions for the improvement of healthcare services were mentioned by 44 patients (53.7%) in the free text box. Most importantly better informational support was stated (18.3%), optimizations in the healthcare setting (18.3%; e.g., more outpatient services) as well as financial reliefs (17.1%; data not shown). 14.6% desired a broader IBM-specific knowledge from the general healthcare providers and the corresponding adaption of their services, especially for supportive therapies (12.2%; e.g., physiotherapist, general practitioner). Also 11% of the patients pointed out the need for more therapy options in general, for psychological support (9.8%) as well as for drug treatments (8.5%). Improvements regarding the assistive device management was stated from 8.5%.

### Resource consumption

**Fig. 1:**
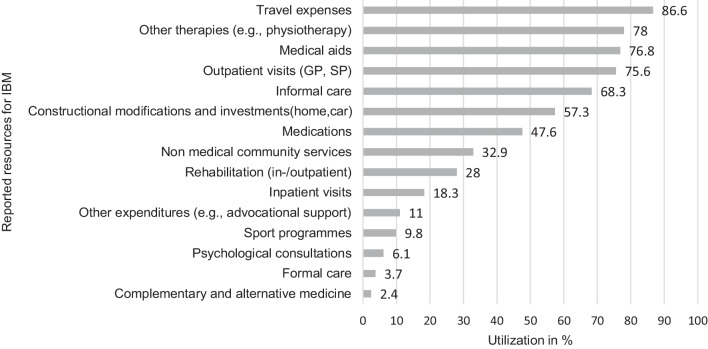
IBM specific resource consumption (n = 82) in percent. *GP* general practitioner, *SP* specialized practitioner

The mean utilization of the medical and nonmedical resources relating to IBM in 1 year is shown in Fig. 1. Most common, 86.6% of the patients reported travel expenses within 1 year to receive health care services. The percentage of travel activities might be higher, as travels without expenditures for the patients were not surveyed. A high utilization was identified for other therapies (78%), of which physiotherapy was intensely utilized (63.4%) followed by occupational therapy (46.3%) and speech therapy (9.8%). Notably, 15.2% of the patients never utilized any of the previously mentioned other therapies, even beyond the recall period.

In our sample, about three in four patients used medical aids (76.8%). Most frequently, 72% of the patients used aids for mobility and show a median of utilizing 2 different mobility aids. Aids for daily living (e.g., raised seats) reported 64.6% of the patients, whereby they show a median of utilizing 3 different aids for daily living. Lastly, aids for care reported 36.6% of the patients (median of 0 of utilized aids for care). Specifically, most of the patients utilized wheeled walkers (46.3%), electric wheelchairs or canes (25.6% each) as mobility aids.

The consumption of outpatient visits (75.6%) was fourfold higher per year than inpatient visits (18.3%). No patient made use of video consultations. Informal care was in place five of the most frequently used resources (68.3%), whereupon the constant presence and support of a caregiver was needed from 22.5% of the patients. The spouses provided most of informal care with a mean of 4 h/day (SD 6.7), considerably different to the means of children (0.3 h/day), grandchildren and other friends or relatives (0.1 h/day each). Full-time informal care was solely utilized from spouses. Moreover, 32.9% of the patients used nonmedical community services for practical support (e.g., home help) and more than half of the patients (57.3%) spent money on constructional modifications of their car or home to increase accessibility.

We identified a consumption of in- or outpatient rehabilitation of 28% in the recall period and almost the same number of patients (24.3%) reported no consumption of rehabilitation ever. In contrast, half of all patients (47.6%) utilized pharmacotherapy relating to their IBM disease (e.g., IVIg or glucocorticoids). Complementary and alternative medicine (e.g., acupuncture), formal care, psychological consultations and sport programmes showed the lowest consumption (2.4%, 3.7%, 6.1%, 9.8%, respectively). Other various disease related expenditures were identified in 11% of the patients.

### Estimated cost of illness

Table 3 shows the estimated annual per capita COI based on the utilized resources. All included patients reported IBM-associated costs. Overall, the average total cost were US$102,682 (95% CI US$82,763–US$123,090). 92.7% of the total COI were direct costs; approximately half of the direct costs were either determined by medical or nonmedical costs. We observed cost for pharmacotherapy as the highest direct medical costs (mean US$30,579; 95% CI US$21,523–US$41,211; 69.3% of direct medical costs and 29.8% of total COI) and formal care as the lowest (mean US$133; 95% CI US$0–US$290; 0.03% of direct medical costs and 0.1% of total COI). The high consumption of informal care causes for 82.9% of direct nonmedical costs (mean US$42,323; 95% CI US$30,119–US$55,191; 41.2% of total COI). Furthermore, some patients reported absent days from work due to inpatient consultations or longer sick leaves (*n* = 4), short-time absences due to outpatient consultations or short-time sick leaves (*n* = 6) and reductions in working hours (*n* = 2). Two patients gave up their jobs due to IBM. In comparison to the direct costs, the low indirect costs (mean US$7,527; 95% CI US$2,005–US$16,349; 7.3% over total COI) represent the large proportion of retired persons in this sample as shown in Table 1.

**Table 3:** Estimated annual COI per patient in € and US$ for the reference year 2021 (n = 82)

	Mean ± SD in € per year	Mean ± SD in US$ per year	% of total COI
Outpatient consultations^a^	830 ± 1699	1122 ± 2295	1.1
Inpatient treatment	1773 ± 5426	2396 ± 7332	2.3
Rehabilitation (in-/outpatient)	769 ± 1673	1039 ± 2261	1.0
Pharmacotherapy	22,629 ± 33,430	30,579 ± 45,176	29.8
Other therapies (e.g., physiotherapy)^b^	3496 ± 3,231	4725 ± 4,366	4.6
Medical aids	3048 ± 6,133	4119 ± 8,287	4.0
Formal care	99 ± 535	133 ± 723	0.1
Total direct medical costs	32,645 ± 36,920	44,115 ± 49,892	43.0
Informal care	31,319 ± 47,880	42,323 ± 64,703	41.2
Nonmedical community services	2916 ± 8448	3941 ± 11,416	3.8
Constructional modifications and investments (home, car)	3062 ± 9543	4138 ± 12,896	4.0
Other expenditures (e.g., advocational support)	33 ± 160	44 ± 216	0.0
Travel expenses	440 ± 745	594 ± 1,007	0.6
Total direct nonmedical costs	37,770 ± 52,189	51,041 ± 70,525	49.7
Total direct costs	70,415 ± 63,595	95,155 ± 85,939	92.7
Total indirect costs	5,570 ± 27,409	7,527 ± 37,040	7.3
Total COI	75,985 ± 67,391	102,682 ± 91,069	100.0

*COI* cost of illness, *SD* standard deviation. Percentages may not total 100 due to rounding

^a^Outpatient consultations include physician visits (specialized and general practitioners) and psychological support

^b^Other therapies include physiotherapy, occupational therapy, speech therapy, complementary and alternative medicine and sport programmes

COI were calculated in euros and converted into US dollars (1US$ = 0.74€; reference year 2021) [48]

**Fig. 2:**
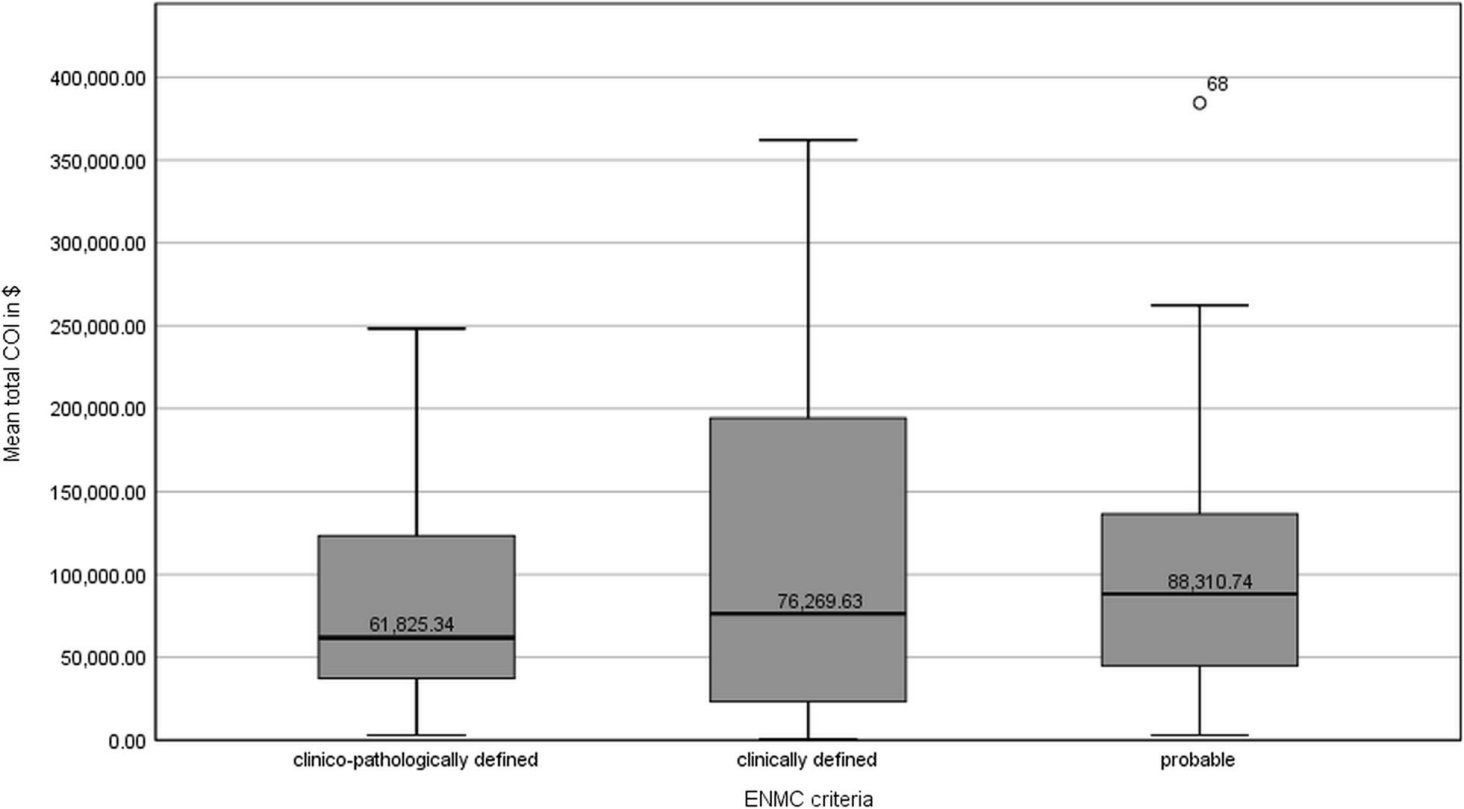
Mean total COI per patient in US$ 2021 depending on ENMC criteria (n = 82). *COI* cost of illness

IBM patients and their caregivers are confronted with a complex care situation. To better understand potential factors that affect the cost and identify differences, we further analysed the total COI exploratively (Table 4). Most significantly married patients (mean US$111,915; 95% CI US$90,939–US$132,778) showed higher total cost (*p* < 0.01) than their unmarried peers (mean US$53,673; 95% CI US$23,541–US$94,051). In addition, we found significant differences (*p* < 0.05) regarding the employment status of the patients: self-employed patients or non-working patients due to IBM had higher total COI than employed patients. A positive correlation was obtained between total COI and the degree of disability (ρ = 0.311; *p* < 0.05), physical function (sIFA) (ρ = 0.175), comorbidity (SCQ-D) (ρ = 0.098) and age (ρ = 0.034). The disease duration correlated negatively (ρ = − 0.118), but not significantly (*p* = 0.294), with the total cost per capita. Despite patients diagnosed with a probable IBM had the highest median of total COI (US$88,311) within the subgroup analysis of the ENMC criteria, the cost are most widely distributed in patients with a clinically defined IBM (Fig. 2). Nevertheless, for most of the analysed variables there were no statistically significant differences and only mild or moderate correlations regarding the total COI.

**Table 4:** Differences and correlations of sociodemographic and disease specific variables regarding the total COI

	N	Mean €	SD	*P*-value (corr. *p*-value^a^)
Age groups				0.728
< 65	19	81,141	14,802	
65–69	13	61,123	16,648	
70–74	20	64,392	12,425	
75–79	15	80,464	17,010	
> 80	15	93,311	23,845	
Age				0.765
Sex				0.946
Female	18	64,697	9521	
Male	64	79,159	9145	
ENMC criteria				0.906
Clinico-pathologically defined	36	67,785	8839	
Clinically defined	28	83,363	15,161	
Probable	18	80,905	17,271	
sIFA				0.118
SCQ-D				0.383
Degree of disability				0.012*
Disease duration (since first symptoms)				0.294
Wheelchair use				0.125
Yes	31	85,033	11,376	
No	51	70,484	9771	
Health insurance				0.107
Privat	26	64,489	8096	
Statutory	54	93,602	15,194	
Satisfaction with healthcare				0.153
Satisfaction with health insurance				0.891
Financial difficulties due to IBM				0.196
Not	32	52,529	7527	
A little	24	89,042	13,581	
Quite a bit	14	83,428	22,567	
Very	9	95,309	27,192	
Care level				0.108
No care level	30	62,153	10,969	
Care level 1	0			
Care level 2	18	63,045	11,568	
Care level 3	20	70,577	12,452	
Care level 4	9	140,824	33,829	
Care level 5	5	110,475	37,284	
Housing conditions				0.133
Flat	28	76,105	12,980	
House	47	81,245	9903	
Home with professional support^b^	7	40,185	20,814	
Marital status				0.005*
Single, widowed, divorced	13	39,718	15,779	
Married	69	82,817	8111	
Employment status				0.049*
Retired	65	74,811	8385	
Non-working due to IBM	2 (employed)^c^	167,552	2382	0.026*^a^ (0.154)
Employed	9 (self-employed)^c^	44,690	14,756	0.032*^a^ (0.191)
Self-employed	5	119,487	33,740	
Educational level				0.274
Medium	46	79,723	9503	
High	33	67,536	11,404	
BMI				0.984
Underweight	0			
Normal weight	37	74,126	10,677	
Overweight	36	79,059	11,773	
Obese	8	78,784	25,602	

*COI* cost of illness, *SD* standard deviation

*p < 0.05

^a^*p*-values after Bonferroni correction are additionally reported in brackets for pairwise comparison of variables showing significant differences in total COI

^b^Including home for the elderly, assisted living and nursing home

^c^Variables with significant pairwise comparisons

In addition, the total national COI were 31.6 million € up to 158.1 million € (US$42.7 million–US$213.7 million; Table 5). The huge range of the total national COI is due to the heterogenous prevalence estimates mentioned in the background. Thus, this ranges highlight realistic ranges of the possible total disease burden in IBM for Germany.

**Table 5:** Overall annual COI for Germany in € 2021

	Mean in € per year, per capita*	Prevalence estimate (Orphanet)^a^	Prevalence estimate (Best estimate Germany)^b^	Total national COI (Orphanet)^a,c^	Total national COI (Best estimate Germany)^b,c^
Total direct medical costs	32,645	5/1,000,000	25/1,000,000	13,586,380	67,931,898
Total direct nonmedical costs	37,770			15,719,331	78,596,654
Total direct costs	70,415			29,305,710	146,528,552
Total indirect costs	5570			2,318,154	11,590,770
Total COI	75,985			31,623,864	158,119,322

*COI* cost of illness

*Data from our COI survey (see Table 3)

^a^Prevalence estimate with European data according to Orphanet Code 611 [41]

^b^Best prevalence estimate according to Callan et al. [6]

^c^Assumed German population in 2021: 83 Mio. (rounded) [78]

## Discussion

This is the first study to estimate the IBM related indirect and direct costs in Germany. We estimated the mean total per capita COI at US$102,682 (95% CI US$82,763–US$123,090) in the reference year 2021. So far, only top-down approaches have been used to estimate IBM related cost, all with US claims data and without patient participation [18, 20, 53]. García‑Pérez et al. suggested that almost 57% of COI studies in rare diseases have not included informal care costs as well as 40% have neither reported direct nonmedical costs nor indirect costs (32%) [25]. We used self-reported questionnaires in our empirical study to comprehensively identify IBM related cost in the German healthcare setting. As previous COI studies have not included all direct and indirect cost components, it was not surprising that the reported annual mean per capita cost in IBM were all suggested lower as US$12,464 [53], US$33,259 [18] and US$44,838 [20], respectively. Stratified cost data for IBM are lacking in studies estimating the COI within IIM as a disease group. Therefore, a study with claims data from Canada reported US$4,099 per patient in polymyositis (PM) and dermatomyositis (DM) [21]. Whereas a cohort study with claims and registry data from Sweden reported €21,639 in the first year after IIM diagnosis and €12,796 at 5-year follow-up, without indicating the included number of IBM patients [54]. Compared to our previous COI studies in other NMD, we identified approximately 2- to fourfold higher per capita COI in IBM than in Becker muscular dystrophy (BMD; mean €39,060) [33] and Charcot-Marie-Tooth neuropathies (CMT; mean €17,427) [31]. Our estimated per capita cost are similar to these of Duchenne muscular dystrophy (DMD; mean €78,913) [33] and spinal muscular atrophy (SMA; mean €70,566) [32].

The large standard deviations in our analysis are also found in other COI studies in IBM [18, 20]. Furthermore, we did not find much significant differences or correlations within the analyses of possible cost driving variables. This might represent medical and nonmedical practice variations or heterogenous disease courses and trajectories [16, 55, 56]. Environmental (e.g., access to health promotion, social support) and personal modifiable risk factors (e.g., disuse or overuse of healthcare resources, therapy adherence) may also be attributed to influence outcomes on the societal and the individual patient level [57]. Therefore, our results yielded some unique evidence about the suggested use of medical and nonmedical resources due to PRO and PRE data from patients in the German IBM patient registry (www.ibm-register.de).

Comparing our estimated direct medical costs to the mean healthcare cost per person in Germany in the year 2021, the cost for IBM are sevenfold higher [58]. In our findings, pharmacotherapy accounts for 69.3% of the total direct medical costs, most importantly due to the high treatment costs of IVIg. This is consistent with studies assessing the COI in chronic inflammatory neuropathies, where IVIg was the main determining cost factor [59, 60]. Current national guidelines in the management of IBM recommend treatment attempts with IVIg for 6 months and continuing the treatment in case of improved or stabilized outcomes [14]. By contrast, 19.5% of our included patients have never received IVIg treatment, showing a mean disease duration in these patients of 6.2 years (range 1–13 years). Of the 41.5% utilizing IVIg treatment in the recall period, the treatment duration ranged from 0.4 to 10 years. A study from Sweden observed that 82% of the IBM patients ever tried immunomodulating treatment [61]. Nevertheless, there is yet no international consensus on the effectiveness of IVIg treatment in IBM, although positive treatment responses are observed [13, 62]. In addition, inconsistent cost coverage of IVIg depending on health system and health insurance could increase uncertainties that promote practice variations in routine clinical practice and heterogenous patient outcomes.

The other essential part of the recommended treatment attempts in IBM is physiotherapy [14]. Only 63.4% of our included patients reported a utilization of physiotherapy in the recall period. Interruptions of the treatment during the COVID-19 pandemic could be a reason for a general underuse of healthcare resources [63]. Gupta et al. identified in a sample of myositis patients (*n* = 608) a disruption of physiotherapy in 35.2% during COVID-19 [64]. However, in 2014 Hiscock et al. [65] already identified that 21% of the surveyed IBM patients had never utilized physiotherapy and 31%, who had utilized physiotherapy, but stopped 1 year after diagnosis. In Germany, individual applications from IBM patients to their statutory health insurances are so far required to get a claim for long-term prescriptions of physiotherapy [66]. This could be a possible barrier for continuous supportive therapies.

Interestingly, no video consultations had been utilized in the recall period, although other studies in myositis observed up to 69.9% using remote services during the COVID-19 pandemic [64]. While the evidence is limited regarding the effectiveness of telemedicine in rheumatology and neurology, the German Society for Rheumatology also recommends telemedicine in the post-pandemic healthcare, e.g. for screenings and follow-up controls [67].

In interpreting our findings of the SCQ-D, it appears relevant to ensure a screening for untreated and burdensome comorbidities along the patient journey, especially for back pain, arthritis, ulcer or stomach disease and depression. Such misallocations of healthcare resources could lead to increased societal cost and a higher disease burden for patients and caregivers [68]. In other studies, comorbidities were higher in IBM patients than in controls (random selection of individuals with more than one healthcare encounter in 1 year) or were identified to be a relevant cost determinant, e.g. depression [18, 60]. Although 6.1% of our patient group utilized psychological support, that is similar to a sample from the US where 8 of 96 patients (8.3%) used emotional support or counselling services within a recall period of 6 months. Nearly the same proportion in our study (9.8%) requested an expansion of professional psychological services.

The utilization of medical aids (mean US$4,119, SD US$8,287) accounts for 9.3% of the direct medical costs. In our sample mobility aids were utilized a little more (72%) than the approximate one-third in the study of DeMuro et al.[69] Capkun et al. summarised the mean costs for medical aids of US$9,975 (SD US$39,417) per year since diagnosis, whereas our data shows lower costs with smaller standard deviations (mean US$4,119, SD US$8,287). In more detail, the estimated costs for inpatient and outpatient consultations are also approximately 51–94% higher in previous studies from the US [18, 20]. At this point it is necessary to consider the different unit prices as well as medical practice in the respective healthcare systems and not jump to conclusions.

Further, the cost analysis revealed that informal care is a major cost factor of direct nonmedical costs and total COI, suggesting a high caregiver burden for the spouses. The significantly higher cost in married patients could explicate, that those patients without a spouse experience less support in everyday life. The utilization of unpaid caregiver support is slightly higher (68.3%) in our study than in an US sample (60%) [19]. In general, other community services seem to be selective add-on services in approximately one third of the patients, almost the same as in the abovementioned cross-sectional study [19]. In contrast to our previous COI studies in SMA, DMD, BMD and CMT the estimations for informal care costs in IBM are considerably higher: 1.5-fold higher than in DMD up to 5.3-fold higher than in CMT [31–33].

Additionally, we identified that the costs for constructional modifications and other expenditures seem to be less relevant in contrast to other cost components. Therefore, it is important to discuss that these spendings are not distributed equally along the progression of the disease, different from what we had originally assumed in our methods. Unique expensive modifications, mostly out-of-pocket spendings, could cause a very high financial burden for individuals at some point in time. Notwithstanding the high educational levels and proportion of private insurance, nearly one third perceived quite a bit to very high financial difficulties due to IBM.

So far, no data on indirect costs in IBM exist beyond our present study (mean US$7,527, SD US$37,040). When examined critically, the human capital approach in general leads to an overestimation of indirect costs, but it does not include priced productivity losses of non-employed persons (retiree, non-workers, househusbands or housewives) [34, 35, 70]. Transferring this to our sample, the indirect costs may even have been underestimated, as 80.2% are retired.

In summary, we conclude that the annual societal cost in German IBM patients are between €31.6 million (US$42.7 million) up to €158.1 million (US$213.7 million), depending on the assumed prevalence estimate. Lindgren et al. identified a prevalence of 32 per million inhabitants in Sweden over a 33-year period, which is higher than our tentative assumptions for Germany in this study (25 per million) [61]. This highlights the impact of uncertain and limited epidemiological prevalence data for Germany on future estimate efforts.

In contrast to our previous COI studies, the societal burden in IBM is suggested lower as in patients with CMT, but it is in the range of patients with BMD, DMD or SMA [31–33]. IBM is often described as a disease in the elderly, thus the caregiving and also ageing spouses carry a profoundly burden, illustrated in the high costs for informal care in this study. On this basis, the qualitatively gathered patients’ voices for more information, support services and research activities in the field of pharmaceutical therapies could have a relevant impact for improving both patient and caregiver burden. However, the patients were rather satisfied with their healthcare and insurance services, but this PREM might not represent actual gaps in healthcare provision.

The main strength of this COI registry-study is the comprehensive estimation of direct and indirect costs in IBM as well as the description of the actual resource utilization, taking PRO and PRE into account. Thus, our study provides evidence to better understand the economic consequences and care situation of IBM for society, payers and eventually for patients. Further, our obtained response rate of 74% was the highest ever within our previous COI studies in other NMD and higher than in any other COI study in NMD [31–33, 71]. This was surprising, as our questionnaire design was very extensive, demonstrating either a high patient motivation or pointing out unheard needs in IBM, along with a strong commitment and close bond between patients and the registry curator. Despite our sample is smaller than previous COI studies in IBM, the patients’ characteristics are similar.

This study has some limitations that should be noted. Firstly, the utilized resources were reported from patients and therefore could cause a recall bias. Even minor patient-reported inaccuracies regarding the IVIg dosing and quantity of treatment could influence cost estimation the most due to the high unit prices. Secondly, there could be a selection bias. As our sample was recruited from a patient-registry, this could imply in general more dedicated patients. In addition, the sample included more patients with a higher educational level and private health insurance than in the general German population. Although IBM is three times more likely to be diagnosed in men than females [72], our sample with 78% males could slightly underrepresent female patients. Thirdly, we observed heterogeneity in our data, though this is not uncommon in small samples with rare diseases. For this reason, we also presented the results after Bonferroni correction, a conservative approach for heterogenous data with large standard deviations [73]. Fourthly, we have extrapolated the mean costs per patient for 1 year according to the reported mean resource utilization in the recall periods (3, 6, 12 and 24 months) and then multiplied the average costs per patient for the overall cost assessment. We have not made further extrapolations based on patient characteristics. Fifthly, we used minimum cost estimates, therefore an underestimation of the actual economic burden is likely. Our recall period comprised the COVID-19 pandemic eventually limiting suggestions for the actual resource utilization outside pandemic periods.

Our results provided deeper insights into the actual care situation of IBM patients and their families. The applied PROMs and PREMs were selected to contrast the estimated COI into the patient-relevant everyday life setting. Taking the different resource utilizations into account, that have been identified by comparing our results from Germany to the US context, this could have important implications for the development of international guidelines in IBM. Whereas more inpatient treatment was identified as important cost driver in the US, an outpatient tendency was observed in our sample [20].

## Conclusion

In conclusion, our study analysed the direct as well as indirect costs in IBM from a societal perspective in the German healthcare setting and stressed the need of accurate epidemiological data. Furthermore, our applied PROMs and PREMs offered new starting points for in-depth health services research in IBM. Our results demonstrate the financial burden of IBM, that is comparable to other severe NMD like SMA, DMD or BMD. Healthcare resources are finite, also the tremendous governmental healthcare spending during the COVID-19 pandemic and decreased gross domestic products worldwide since 2020 intensify the relevance of comprehensive COI studies to provide transparency for efficient healthcare spending from governments, private households and the medical industry. Our study provides transparency in the actual care delivery and the related resource consumption pattern. This will hopefully not only increase the visibility of unmet IBM care needs, but also highlight disease related consequences for the mostly unpaid caregiver due to the vast informal care. Responsive healthcare systems are needed to ensure continuous access to supportive therapies and counselling in this progressive disease.

### Supplementary Information

**Additional file 1**. Applied unit prices for the COI calculation using the reference year 2021.

## Data Availability

The raw dataset is not publicly available to preserve individuals’ privacy under the European General Data Protection Regulation. Only excerpts of the datasets used during the current study are available from the corresponding author (klaus.nagels@uni-bayreuth.de) on reasonable request.

## Abbreviations

BMD: Becker muscular dystrophy
CI: Confidence interval
CMT: Charcot–Marie–Tooth neuropathies
COI: Cost of illness
DMD: Duchenne muscular dystrophy
ENMC: European neuromuscular centre
FDA: United States Food and Drug Administration
IBM: Inclusion body myositis
IIM: Idiopathic inflammatory myopathy
IQWiG: Institute for Quality and Efficiency in Health Care
IVIg: Intravenous immunoglobulins
HRQoL: Health-related quality of life
NMD: Neuromuscular diseases
US: United States
PRO: Patient-reported outcome
PROMs: Patient-reported outcome measures
PRE: Patient-reported experience
PREMs: Patient-reported experience measures
SCQ-D: German Self-Administered Comorbidity Questionnaire
sIFA: sIBM Physical Functioning Assessment
SMA: Spinal muscular atrophy
