# Supplementary material for: Cost of illness in inclusion body myositis: results from a cross-sectional study in Germany
Source: Orphanet J Rare Dis. 2023 Oct 25;18:337. doi: 10.1186/s13023-023-02902-3 (PMC10601274; doi:10.1186/s13023-023-02902-3)
Supplement: Supplementary file 1 — Additional file 1. Applied unit prices for the COI calculation using the reference year 2021. [file 13023_2023_2902_MOESM1_ESM.docx]

**Additional file 1**

Applied unit prices for the COI calculation using the reference year 2021.

| **Resource** | **Value** | **Unit** | **References** |
| --- | --- | --- | --- |
| Outpatient consultations |  |  |  |
| General practitioner | 27.42 | €/visit | [1] |
| Neurologist | 64.72 | €/visit | [1] |
| Orthopaedist | 33.92 | €/visit | [1] |
| Internist | 87.36 | €/visit | [1] |
| Surgeon | 56.27 | €/visit | [1] |
| Ear, nose and throat specialist | 40.88 | €/visit | [1] |
| Other therapies |  |  |  |
| Physiotherapy | 22.41 | €/visit | [1] |
| Occupational therapy | 56.27 | €/visit | [1] |
| Speech therapy | 62.76 | €/visit | [1] |
| Complementary and alternative medicine^a^ | 47.14 | €/visit | [1] |
| Inpatient consultations |  |  |  |
| General medical unit | 1,054.30 | €/day | [1, 2] |
| Intensive care unit | 1,816.44 | €/day | [1, 2] |
| Rehabilitation |  |  |  |
| Inpatient | 147.25 | €/day | [1] |
| Outpatient | 74.63 | €/day | [1] |
| Medical devices^b^ |  |  | [1, 3] |
| Care at home |  |  |  |
| Basic care | 34.62 | €/hour | [1, 4] |
| Care at outside home, short-term |  |  |  |
| Care level 1 | 78.89 | €/day | [1] |
| Care level 2 | 88.56 | €/day | [1] |
| Care level 3 | 100.31 | €/day | [1] |
| Care level 4 | 112.13 | €/day | [1] |
| Care level 5 | 118.47 | €/day | [1] |
| Care at outside home, semi-residential  (day / night)^c^ |  |  |  |
| Care level 1 | 52.20 / 41.37 | €/day | [1] |
| Care level 2 | 58.03 / 45.97 | €/day | [1] |
| Care level 3 | 62.70 / 50.57 | €/day | [1] |
| Care level 4 | 67.41 / 55.21 | €/day | [1] |
| Care level 5 | 71.15 / 59.78 | €/day | [1] |
| Informal care | 19.05 | €/hour | [1, 5] |
| Indirect costs^d^ |  |  |  |
| Gross salary (men / women) | 4,275 / 3,699 | €/year | [6] |
| Medications |  |  | [7] |

^a^If private payments have not been specified.

^b^If medical device could not be classified according to the medical device hierarchy in the REHADAT system; a German information system comprising data about medical companies, support organizations for disabled people as well as a hierarchical classification of medical devices [8].

^cf^In 2017, the definition of the need for care was revised in Germany. The extent of benefits from the German statutory care insurance are based on an individual score within six life domains of a person. A higher care level indicates a worse state of independence and capabilities [9].

^d^If self-reported wage was missing.

**References**

1. Bock J-O, Brettschneider C, Seidl H, Bowles D, Holle R, Greiner W, König HH. Ermittlung standardisierter Bewertungssätze aus gesellschaftlicher Perspektive für die gesundheitsökonomische Evaluation. Das Gesundheitswesen. 2015;77:53–61.

2. Krauth C, Hessel F, Hansmeier T, Wasem J, Seitz R, Schweikert B. Empirische Bewertungssätze in der gesundheitsökonomischen Evaluation—ein Vorschlag der AG Methoden der gesundheitsökonomischen Evaluation (AG MEG). [Empirical standard costs for health economic evaluation in Germany—a proposal by the working group methods in health economic evaluation]. Gesundheitswesen. 2005;67:736–46. doi:10.1055/s-2005-858698.

3. Müller R, Rothgang H, Glaeske G. BARMER GEK Heil- und Hilfsmittelreport 2015. 2015. https://www.barmer.de/resource/blob/1027106/168499ade7c25bf26b6383a14c634f7e/barmer-gek-heil-und-hilfsmittelreport-2015-data.pdf. Accessed 14 Oct 2022.

4. Verband der Ersatzkassen e. V. Landesvertretung Bayern (vdek). Vereinbarung über Gebühren für Leistungen der häuslichen Krankenpflege (§ 37 SGB V) vom 09.02.2021 mit Gültigkeit für ab 01.04.2021 – 31.12.2022 erbrachte Leistungen (AC/TK 31 02 491). 2021. https://www.vdek.com/LVen/BAY/Vertragspartner/pflege-2/haeusliche-krankenpflege/_jcr_content/par/download_1768783445/file.res/Geb%c3%bchrenvereinbarung%20der%20Wohlfahrtsverb%c3%a4nde%20ab%20dem%2001.04.2021.pdf. Accessed 14 Oct 2022.

5. Bundesministerium für Arbeit und Soziales. Mindestlöhne und Mehrurlaub in der Pflege: Fragen und Antworten. 2020. https://www.bmas.de/SharedDocs/Downloads/DE/Publikationen/a763-ml-pflegebranche-broschuere-pdf.pdf?__blob=publicationFile&v=1. Accessed 14 Oct 2022.

6. Statistisches Bundesamt (DeStatis). Verdienste und Verdienstunterschiede: Entwicklung der Bruttomonatsverdienste. 2022. https://www.destatis.de/DE/Themen/Arbeit/Verdienste/Verdienste-Verdienstunterschiede/Tabellen/lange-reihe-deutschland.html?view=main[Print]. Accessed 25 Apr 2022.

7. CompuGroup Medical Deutschland AG. LAUER-TAXE® – reliable pharmaceutical information for all drugs and contracts registered in Germany. 2022. https://www.cgm.com/deu_de/produkte/apotheke/lauer-taxe-en.html. Accessed 14 Oct 2022.

8. Instituts der deutschen Wirtschaft Köln e.V., Bundesministerium für Arbeit und Soziales (BMAS). Well informed with REHADAT: Information on participation and inclusion in working life. 2022. https://www.rehadat.de/en/. Accessed 14 Oct 2022.

9. Das Elfte Buch Sozialgesetzbuch (SGB XI) – Soziale Pflegeversicherung – Art. 1 des Gesetzes vom 26. Mai 1994, BGBl. I S. 1014, 1015), das zuletzt durch 7e des Gesetzes vom 27. September 2021 (BGBl. I S. 4530) geändert worden ist.
